# From bench to patient: model systems in drug discovery

**DOI:** 10.1242/dmm.023036

**Published:** 2015-10-01

**Authors:** Matthew D. Breyer, A. Thomas Look, Alessandra Cifra

**Affiliations:** 1 Eli Lilly and Company, Indianapolis, IN 46285, USA; 2 Department of Pediatric Oncology, Dana-Farber Cancer Institute and Harvard Medical School, Boston, MA 01864, USA; 3 Disease Models & Mechanisms, The Company of Biologists, Bidder Building, Station Road, Histon, Cambridgeshire, CB24 9LF, UK

## Abstract

Model systems, including laboratory animals, microorganisms, and cell- and tissue-based systems, are central to the discovery and development of new and better drugs for the treatment of human disease. In this issue, *Disease Models & Mechanisms* launches a Special Collection that illustrates the contribution of model systems to drug discovery and optimisation across multiple disease areas. This collection includes reviews, Editorials, interviews with leading scientists with a foot in both academia and industry, and original research articles reporting new and important insights into disease therapeutics. This Editorial provides a summary of the collection's current contents, highlighting the impact of multiple model systems in moving new discoveries from the laboratory bench to the patients' bedsides.

## Abstract (precis)

**Drug Discovery Collection:** This Editorial introduces the new DMM Special Collection entitled ‘From bench to patient: model systems in drug discovery’, providing a summary of its contents and highlighting the impact of multiple model systems in moving new discoveries from bench to patient.

## Introducing the Special Collection

Despite widespread dedicated research efforts from academia and industry that have driven the discovery of treatments for many diseases, society continues to face substantial unmet medical needs for numerous health issues afflicting humanity. Recent publications have focused on the differences between human diseases and animal models of disease (Seok et al., 2013), and have noted in some cases the failure of the latter to predict therapeutic efficacy (Suckling, 2008; Pippin, 2014). Because of this, some have advocated abandoning animal studies and focusing on clinical trials in human patients (Shanks et al., 2009); however, the fact remains that the ethical and monetary hurdles to primary screening of molecules in humans are insurmountable. Medical research remains dependent on model organisms and systems for establishing efficacy prior to the design of clinical trials. New approaches to validate cellular and animal models of disease that harmonise their behaviour with human disease are now being developed. These advances include reverse translation of human monogenetic disease to establish homologous cell-based or animal disease models, the use of induced pluripotent stem cells, and molecular fingerprinting of diseased tissues in human versus animal models (Shultz et al., 2012; Trounson et al., 2012; Hodgin et al., 2013; Seok et al., 2013; Scheer and Wolf, 2014). Many of these advances are reviewed and exemplified in the content of this Special Collection. As the molecular basis of disease pathogenesis is elucidated, the utility of these faithful disease models will only increase.

Over the past few years, *Disease Models & Mechanisms* (DMM) has witnessed an increase in high-quality research submissions reporting advances in drug discovery and development. We continue to be committed to supporting and inspiring scientists in this research area, and we appreciate the importance of further promoting communication between basic researchers, clinicians and scientists from both academia and industry. We are therefore compiling an online and printed Special Collection to highlight the pertinent reviews and research articles newly or recently published in DMM on the use of model systems in drug discovery. In this collection, one will find research papers reporting the use of a variety of model systems to test or develop new drugs; review articles that summarise and critically discuss different approaches to disease treatment; as well as Editorials and interviews from scientists who have contributed to translating research discoveries into patient therapies. Below, we provide a summary of all the articles in the collection so far, highlighting the translational impact of each of the research papers in particular. We also describe some of the articles that will be added to the collection in the coming months.

**Figure:**
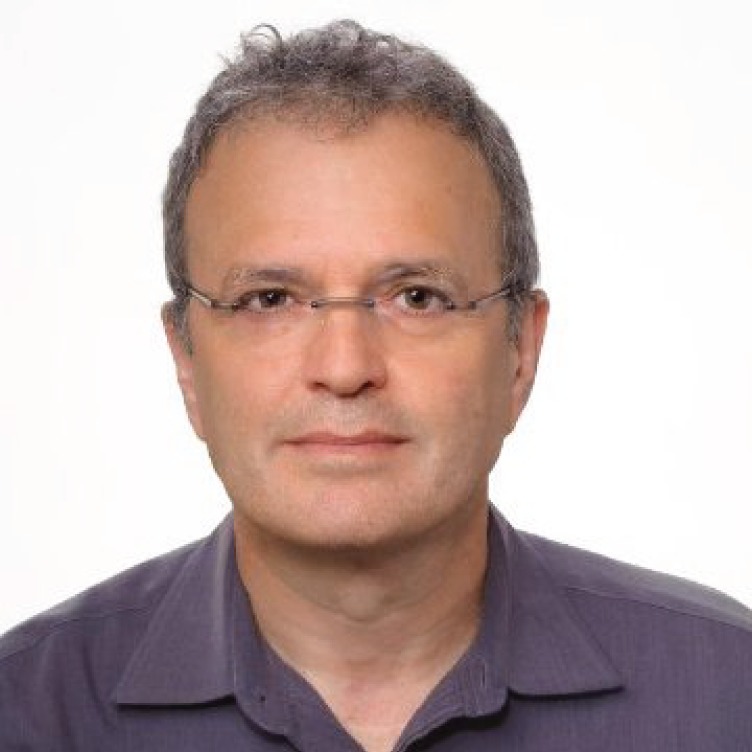
**Matthew D. Breyer**

**Figure:**
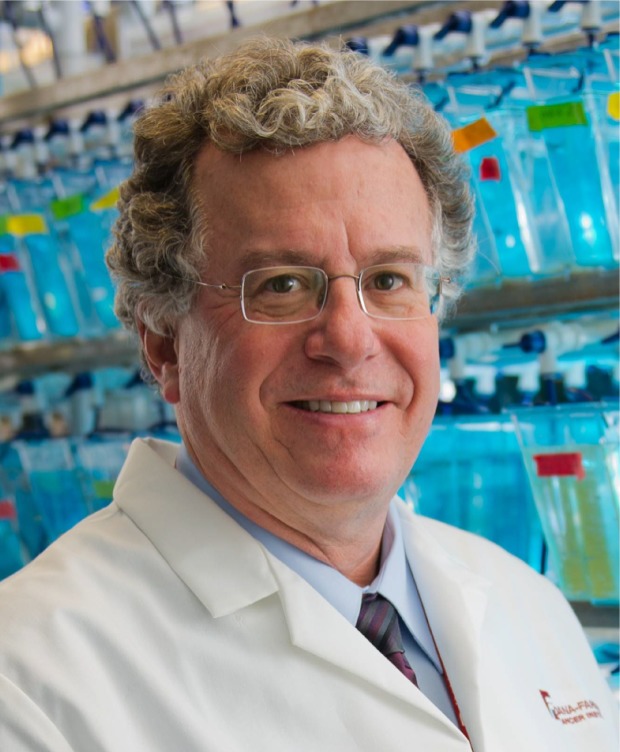
**A. Thomas Look**

## Drug discovery discussed: Editorials, interviews and reviews

At the forefront of the collection is an Editorial from **Dominic Wells** (Wells, 2015), who discusses ways to improve translational studies through better animal experiments. Dominic provides examples of good laboratory practice and guidelines that are being implemented in the rare disease field, an approach that could be adopted by other disease fields to achieve greater success in taking animal data to clinical trials. In an ‘A Model for Life’ interview, **Geoffrey Smith** (Smith, 2015), current Managing Director of Mars Ventures and past senior member of the DMM editorial team, tells us how he developed his incredibly varied career, from the desk of law school to the leadership of healthcare companies, via several academic positions. As well as sharing his personal journey, Geoff describes how academia and industry can fruitfully meet to drive the successful discovery of new therapeutics. Next, in the first newly published Review of this collection, **Owen Sansom and colleagues** (Gopinathan et al., 2015) highlight the utility of genetically engineered mouse models (GEMMs) for the preclinical testing of pancreatic cancer therapies, prior to their transition to the clinical arena. In this Review, the authors describe the potential of GEMMs for predicting clinical outcomes in humans, and critically discuss their limitations as well as opportunities for improvement.

We also present a selection of review articles published in DMM in 2014 and 2015 showcasing the use of different animal and cellular systems in the discovery of therapies for multiple human conditions. As part of this ‘best of’, **Jean-Paul di Rago and collaborators** (Lasserre et al., 2015) explore yeast as a system to model mitochondrial dysfunction and show how genetic and chemical screens in this microorganism can unravel therapeutic targets for mitochondrial disorders. In two articles, **Dongsheng Duan's group** (McGreevy et al., 2015) and **Anand Swaroop's group** (Veleri et al., 2015) review the use of mouse models for developing innovative gene therapy for the treatment of Duchenne muscular dystrophy and inherited retinal diseases, respectively.

Next, **Catherine D. McCusker and David M. Gardiner** (McCusker and Gardiner, 2014) describe the salamander as an exceptional model to optimise cell-based regenerative therapies in humans. Cell-based therapies are also the focus of the Review by **Dennis O. Clegg and colleagues** (Forest et al., 2015), who highlight the potential of stem-cell-based therapies for treating age-related macular degeneration and also describe currently available cellular systems for screening drug candidates for this disease. In addition, **Michaela Sharpe and Natalie Mount** (Sharpe and Mount, 2015) and **Gerald T. Nepom's group** (Smilek et al., 2014) explore approaches to manipulate immune cells *ex vivo* as a promising therapeutic strategy for cancer and autoimmune disease, respectively.

Last but not least, the zebrafish has recently emerged as an increasingly prominent model in translational research and drug discovery [see our popular poster article by **Jennifer B. Phillips and Monte Westerfield** (Phillips and Westerfield, 2014)]. You can read about the potential of zebrafish xenotransplantation in cancer drug screening in the Review from **Jason N. Berman and colleagues** (Veinotte et al., 2014), whereas the use of zebrafish in the search for therapies against human cardiovascular disease and motor neuron disorders is eminently discussed in the articles from **Aarti Asnani and Randall T. Peterson** (Asnani and Peterson, 2014) and **Pierre Drapeau's group** (Patten et al., 2014), respectively.

Articles coming soon include an ‘At a Glance’ poster article in which **Ken Rhodes** illustrates the typical drug development pipeline, from the identification of a promising compound to its approval and market entry. In addition, a Review article from **Chad Cowan's group** will discuss the benefits of using genome editing in human stem cells to model disease-associated gene mutations and will then describe how these genetically modified stem cells can be used as a platform to screen and identify new therapeutic compounds. In another article, **Peter Roy and colleagues** will highlight the uniqueness of *Caenorhabditis elegans* in the identification of novel bioactive molecules in various diseases, including microbial and parasitic infection, cancer, and neurological disorders. They will also stress the untapped potential of this invertebrate and the need to incorporate worm models of human disease into chemical screens to identify new therapeutics. Finally, **Jonathan Himmelfarb's group** will review how the use of three-dimensional kidney microphysiological systems can accelerate drug development by facilitating *in vitro*-to-*in vivo* scaling of a drug candidate from the preclinical phase to clinical trials, and how these systems can enhance patient safety by predicting human toxicity.

Finally, don't miss our selection of ‘A Model for Life’ interviews with **David Rubinsztein** (Rubinsztein, 2015), **Len Zon** (Zon, 2014), **Rick Morimoto** (Morimoto, 2014), **Elaine Mardis** (Mardis, 2014), **Owen Sansom** (Sansom, 2014), **Hans Clevers** (Clevers, 2013), **Ross Cagan** (DMM Editor-in-Chief) (Cagan, 2013) and **George Tidmarsh** (Tidmarsh, 2013), who all have been directly involved in taking drug candidates from the bench to patient.

## Research: model systems in action to discover new drugs

In the research section of this collection, **Jan-Luuk Hillebrands and colleagues** (Poosti et al., 2015) provide an example of how *ex vivo* tissue systems are central to modelling disease and testing drugs. The group use precision-cut kidney slices (PCKSs) prepared from mouse kidneys and apply transforming growth factor beta-1 (TGFβ1) to mimic fibrosis development. They find that TGFβ1 markedly increases the expression of extracellular matrix proteins and of markers of myofibroblast formation, key contributors to renal fibrosis. Notably, application of interferon gamma (IFNγ) or of an IFNγ conjugate counteracts the fibrotic changes. Thus, PCKSs represent a novel tool to model early-onset fibrosis and to screen the efficacy of anti-fibrotic drugs and cytokines *ex vivo* in a multicellular and pro-fibrotic environment, which cannot be replicated in *in vitro* homogeneous cell populations.

*In silico* computational analyses have the potential to speed up the discovery of new drugs by quickly and effectively screening drug candidates. Via *in silico* evaluation of several derivatives of calixpyrroles (macrocyclic compounds capable of binding to anions and neutral substrates), **Marcello Maggiolini's group** (Lappano et al., 2015) discover that the calix[4]pyrrole derivative, C4PY, can act as an antagonist of the G protein-coupled receptor GPER, which mediates stimulatory estrogen signalling in malignant tissues. Interestingly, C4PY elicits inhibitory effects on GPER-induced responses, including protein kinase phosphorylation, gene transcription, cell proliferation and migration in breast cancer cells and in cancer-associated fibroblasts, thus representing a novel compound to consider for targeting GPER in cancer cells. Along the same lines, **Bhagwan Yadav, Tero Aittokallio and colleagues** (Yadav et al., 2015) implement a computational target deconvolution approach to disclose signals of addiction – a phenomenon describing the dependency of certain tumours on one single oncogene for growth and survival – in individual cancer subtypes. Addiction signatures can thus reveal anti-cancer drug targets. The authors apply the target deconvolution approach to more than 100 cancer cell lines and to patient-derived leukaemia cell models, revealing pan-cancer correlations in their addiction signatures. This approach offers functional insights into patient-specific addiction patterns that might lead to clinically actionable, personalised treatments against cancer.

*In vivo* testing is another vital step in the drug discovery and development pipeline. By using obese Zucker rats, a model of genetic obesity, **Antonia Serrano's team** (Decara et al., 2015) evaluate the *in vivo* effects of a novel oleic acid conjugated with an amphetamine derivate (OLHHA) on fatty liver disease. The authors discover that OLHHA reduces both liver lipid accumulation and plasma triglyceride levels. In addition, OLHHA displays a safe pharmacological profile. Thus, this compound could be used to treat non-alcoholic fatty liver disease and other liver complications associated with obesity.

Models based on the transplantation of human cells into a host of another species (xenografts) are a powerful system to study disease-related human alterations in the context of a whole animal. **Jiin-Haur Chuang and colleagues** (Huang et al., 2015) use mouse xenografts to test the *in vivo* effects of the glycolytic inhibitor 2-deoxyglucose (2DG) in suppressing the growth of neuroblastoma, particularly the type showing amplification of the *MYCN* oncogene that has a poor prognosis in children. The group transplant the SK-N-DZ and SK-N-AS human neuroblastoma lines (a *MYCN*-amplified and a *MYCN*-non-amplified neuroblastoma line, respectively) into young mice and monitor tumour growth in response to 2DG administration. The drug is able to reduce tumour size regardless of the status of *MYCN* amplification, and this effect is associated with the downregulation of the metabolic regulators HIF-1α, PDK1 and c-Myc and with a reduction in the number of tumour vessels. 2DG thus represents a potential compound to target the metabolic regulation and angiogenesis of cancer cells in human refractory neuroblastoma.

*p48^Cre^; LSL*-*Kras^G12D^; Cdkn2a^f/f^* (KIC) mice are a well-known preclinical mouse model of aggressive pancreatic ductal adenocarcinoma (PDA). **Thomas M. Wilkie and colleagues** (Ocal et al., 2015) take advantage of a *Kras^G12D^*-dependent marker of PDA, the *Rgs16::GFP* transgene, and use it to trace PDA progression. With this rapid *in vivo* screen, they find that KIC mice treated with gemcitabine plus inhibitors of Axl signalling (an emerging cancer drug target) show fewer tumour initiation sites and reduced tumour size than KIC mice treated with standard-of-care treatments. This is a proof-of-principle drug screen demonstrating the potential of *Rgs16::GFP* as an *in vivo* reporter of PDA progression and drug sensitivity, an approach that could be exploited to identify improved therapeutics also for other cancer types.

Among the research articles published previously in 2015, **Moses Rodriguez and colleagues** (Xu et al., 2015) show that a natural human IgM antibody, capable of binding to neuronal membranes and of supporting axon extension, can attenuate degeneration of neurons and axons in two mouse models of amyotrophic lateral sclerosis. In another article, **Glen T. Prusky and colleagues** (Alam et al., 2015) use mouse models of type-1 and type-2 diabetes and discover that MTP-131, a mitochondrial therapeutic, reverses diabetes-associated visual decline. Given the lack of agents that can treat visual loss in diabetic retinopathy, this compound shows promise to complement existing therapies for this pathology.

Further supporting the utility of zebrafish in high-throughput chemical screening, in an elegant study **Shawn M. Burgess and colleagues** (Gallardo et al., 2015) perform a phenotype-driven screen using transgenic zebrafish that express a fluorescent GFP-labelled marker to track migration of lateral line primordium cells, a cluster of cells that collectively migrate along the zebrafish body axis during development. With this assay, they identify a novel compound, SU6656, which interferes with primordium migration without overt toxicity in zebrafish. Notably, this newly identified compound exerts anti-metastatic activity in two highly aggressive mammary tumour cell lines and in a mouse orthotopic cancer model. Phenotype-driven screens in zebrafish can thus reveal novel targets for therapeutic intervention with a higher success rate and in a more time- and cost-effective manner.

In another research paper, **Tomàs Pinós and colleagues** (de Luna et al., 2015) culture skeletal muscles derived from a knock-in mouse model carrying the most common mutation of the inherited metabolic disorder McArdle disease, which is caused by mutated muscle glycogen phosphorylase (GP-MM). These skeletal-muscle cultures can fully mimic the main biochemical and histological alterations associated with McArdle disease, including glycogen accumulation. In this *in vitro* system, the authors show that sodium valproate, a drug suggested to have therapeutic potential in this disease, reduces glycogen accumulation and also increases the expression of the brain GP isoform. These primary skeletal-muscle cultures can be immortalised and used as a high-throughput screening system for new drugs against McArdle disease.

Animal models are also an incredibly valuable tool to further elucidate the mechanism of action of known drugs. This is exemplified in the study by **Jeffrey L. Neul and colleagues** (Herrera et al., 2015), who use a mouse model of Rett syndrome (RTT) to elucidate the potential of Na^+^-channel- and β-blockers in attenuating long QT (LQT), a common arrhythmia in individuals with RTT. They find that only the Na^+^-channel-blocker phenytoin normalises the LQT arrhythmia in the mice and that, in addition, the drug ameliorates obesity phenotypes, which are often associated with RTT. This suggests Na^+^-channel blockers as a treatment option for RTT-affected individuals with LQT.

This launch issue of the Special Collection highlights the diversity of models and approaches that have come into play to successfully advance the discovery of new drugs for human disease. We hope that this collection further inspires collaboration between academia and industry in translational science. As this is a fruitful and burgeoning area, we will expand this collection with fresh content – including further original research – over the coming months. To keep abreast of new and exciting insights into disease therapeutics using model systems, bookmark the DMM drug discovery collection page at http://dmm.biologists.org/cgi/collection/drugdiscovery and don't forget to subscribe for our email alerts at http://dmm.biologists.org/cgi/alerts/collalert.

## Notes

This article is part of a subject collection on Model Systems in Drug Discovery: from Bench to Patient. See related articles in this collection at http://dmm.biologists.org/cgi/collection/drugdiscovery.
